# Study of Stabilization Mechanism of CO_2_ Foam Flooding System Using Gas-Soluble Nonionic Surfactant and Nano-SiO_2_

**DOI:** 10.3390/molecules31203693

**Published:** 2026-10-15

**Authors:** Jiuzheng Yu, Xiao Qu, Shichun Yu, Yuqin Jia, Ping Yi, Minglei Tian

**Affiliations:** 1 Oil and Gas Technology Institute of PetroChina Changqing Oilfield Company, Xi’an 710018, China; yujzh_cq@petrochina.com.cn (J.Y.); ysc_cq@petrochina.com.cn (S.Y.); jiayuqin_cq@petrochina.com.cn (Y.J.); yip3_cq@petrochina.com.cn (P.Y.); 2 National Engineering Laboratory for Exploration and Development of Low Permeability Oil and Gas Fields, Xi’an 710018, China; 3 College of Chemistry and Environmental Engineering, Yangtze University, Jingzhou 434023, China; tianm086@yangtzeu.edu.cn

**Keywords:** CO_2_ foam flooding, gas-soluble nonionic surfactant, supercritical CO_2_, nano-SiO_2_, drainage half-life

## Abstract

CO_2_ flooding is essential for enhancing oil recovery in low-permeability reservoirs; however, the large viscosity contrast between CO_2_ and crude oil leads to severe gas channeling and low sweep efficiency. In this study, a binary composite system was constructed with a gas-soluble nonionic surfactant (OXO1312) as the foaming core and nano-SiO_2_ as the Pickering stabilizer. The solubility of the surfactants in scCO_2_ was evaluated at 60 °C and 20 MPa using a variable-volume apparatus, the foam performance was assessed by the Waring Blender method, and the foam morphology was observed by optical microscopy. The nonionic surfactants exhibited markedly higher scCO_2_ solubilities (>2 wt%) than the anionic surfactants (<1 wt%), following a clear carbon-chain-length window effect. OXO1312, combining high CO_2_-philicity with an appropriate EO number, was selected as the optimal foaming agent. The addition of 1.5 wt% nano-SiO_2_ increased the drainage half-life of the OXO1312 system by 84% through pronounced Pickering stabilization. The binary system remained stable below 50 °C under moderate salinity, whereas the half-life declined sharply above 70 °C. These findings provide a theoretical basis for the formulation design of CO_2_ foam flooding systems for CCUS-EOR.

## 1. Introduction

CO_2_ flooding is an important technique for enhancing oil recovery (EOR) in low-permeability and ultra-low-permeability reservoirs, and it holds dual strategic significance for safeguarding national energy security and achieving the “Dual Carbon” goals. However, owing to the substantial viscosity contrast between CO_2_ and crude oil (the viscosity of CO_2_ is typically 0.03–0.10 mPa·s, whereas that of in situ crude oil ranges from 1 to 50 mPa·s), the injected CO_2_ is highly prone to channeling along high-permeability pathways or fractures, leading to viscous fingering, low sweep efficiency, and poor displacement performance. Statistics indicate that, in domestic CO_2_ flooding field trials, gas channeling occurs in 30–50% of the wells, severely restricting the large-scale application of this technology.

CO_2_ foam flooding is an effective approach for suppressing gas channeling and expanding the sweep volume. Through its unique rheological behavior, foam can dramatically increase the apparent viscosity of CO_2_ (up to tens to hundreds of times that of pure CO_2_), forcing the displacing fluid to divert into low-permeability zones and thereby achieving deep fluid diversion. Conventional CO_2_ foam systems typically employ water-soluble surfactants as foaming agents, which are pre-mixed with injection water to form a foaming-agent solution before being alternately injected or co-injected with CO_2_ into the formation [1]. However, this injection strategy faces three major problems. First, the foaming-agent solution suffers severe adsorption loss at the solid–liquid interface when injected into low-permeability formations (typically 2–5 mg/g of rock), and water-phase retention causes water-blocking damage that reduces the relative permeability of the oil phase. Second, non-uniform surfactant adsorption on the rock leads to heterogeneous foam generation, making the local foam quality difficult to control. Third, on-site operations require large-scale liquid-preparation equipment and substantial industrial water consumption, limiting applications in water-scarce regions or on offshore platforms [2].

In recent years, the development of gas-soluble surfactants (i.e., foaming agents that dissolve in the CO_2_ phase) has offered a new route to addressing the above issues. These surfactants are co-injected with CO_2_ and, upon reaching deep formations, contact the residual formation water and generate foam in situ [3]. The advantages of this technical route are as follows: (1) the surfactant is injected in an anhydrous form, avoiding water-blocking damage and impairment of low-permeability reservoirs; (2) the surfactant migrates together with the CO_2_ phase and can reach deep micropores that are inaccessible to water-soluble foaming agents; and (3) the in situ foaming triggered upon gas–liquid contact exhibits self-adaptive “water-triggered foaming” behavior, with foam generation precisely localized in regions where the aqueous phase exists, yielding more targeted profile control.

The key to realizing in situ foaming lies in the sufficient solubility of the surfactant in the CO_2_ phase. Existing studies have shown that fluorine-containing surfactants exhibit relatively high solubility in CO_2_, but their widespread application is limited by high cost and potential environmental toxicity. Hydrocarbon- and silicon-containing surfactants offer better environmental friendliness, yet most exhibit limited solubility in CO_2_ [4]. From the perspective of surfactant type, nonionic surfactants have become the preferred choice for CO_2_-soluble foaming agents due to their highly designable molecular structures, relatively low sensitivity to temperature and salinity, and good biodegradability.

However, CO_2_ foams composed of a single surfactant are thermodynamically unstable systems whose decay mainly involves liquid-film drainage, bubble coalescence, and Ostwald ripening induced by gas diffusion, with elevated temperatures and high salinity further aggravating liquid-film instability [5,6]. Therefore, foam stabilizers must be introduced to enhance the mechanical strength and liquid-phase viscosity of the foam films. Existing stabilizers mainly fall into three categories: water-soluble polymers, biomacromolecules, and nanoparticles. Among them, polymer stabilizers can significantly increase the apparent viscosity under fresh-water conditions but exhibit limited thermal and salt resistance, while biomacromolecular stabilizers are susceptible to thermal denaturation and microbial degradation. In contrast, SiO_2_ nanoparticles have become the preferred stabilizer owing to their irreversible interfacial adsorption (with particle desorption energies on the order of 10^3^–10^5^ kT), wide availability of raw materials, low cost, stable physicochemical properties, and environmental friendliness [7]. Studies have shown that 0.7 wt% SiO_2_ nanoparticles can elevate the apparent viscosity of supercritical CO_2_ foam to 80 mPa·s, yield a resistance factor of 200, and refine the bubble size to 20–50 μm [8]. After surface modification with APTES, particle adsorption at the CO_2_/water interface is further enhanced, increasing the foam half-life by up to 33.3% [9]. Combining silica aerogel with SDS reduces the half-life loss to only 6% over 30–140 °C (compared with a 62% loss for SDS alone) while improving the CO_2_ capture efficiency by 75% [10]. Hence, nano-SiO_2_ was selected as the stabilizing component in this study.

Nevertheless, the foaming performance of nonionic surfactants under reservoir conditions remains rather limited, with foam half-lives typically ranging from only a few minutes to a dozen minutes, which cannot meet the long-term foam stability required for field applications [11,12]. Even with the introduction of inorganic stabilizers such as nano-SiO_2_, the system still suffers from particle agglomeration and accelerated liquid-film drainage upon prolonged high-temperature aging, resulting in only limited improvement in the half-life [13]. Although switchable ethoxylated amine surfactants can achieve “CO_2_-philicity-to-foaming” functional switching under CO_2_-induced acidification and form highly viscous CO_2_/water foams at 120 °C under high salinity [14,15], their synthesis routes are complex, and it is difficult to simultaneously balance the CO_2_-philic structure and the foaming performance [16]. Therefore, there is an urgent need to introduce a new class of functional components that combine CO_2_/water interfacial affinity with high-temperature and high-salinity stability, so as to synergistically construct an integrated foam system together with gas-soluble nonionic surfactants and nano-stabilizers.

Against this background, this study employs gas-soluble fatty alcohol polyoxyethylene ether nonionic surfactants as the core of the foaming system and systematically evaluates their dissolution behavior in supercritical CO_2_ using a variable-volume scCO_2_ apparatus. Combined with the Pickering stabilization mechanism, the effects of the molecular structure of the nonionic surfactants (carbon-chain length and number of hydrophilic groups), temperature, aqueous salinity, and nano-SiO_2_ content on the drainage half-life of CO_2_ foam are thoroughly examined. Through visualization of the foam microscopic morphology and its dynamic evolution within pores, the synergistic stabilization mechanism between the gas-soluble nonionic surfactant and nano-SiO_2_ at the CO_2_/water interface and within the foam films is elucidated, with the aim of providing a theoretical basis for the formulation design of CO_2_ foam flooding systems for CCUS-EOR processes.

## 2. Results and Discussion

### 2.1. Solubility Evaluation of Surfactants in scCO_2_

**Figure 1:**
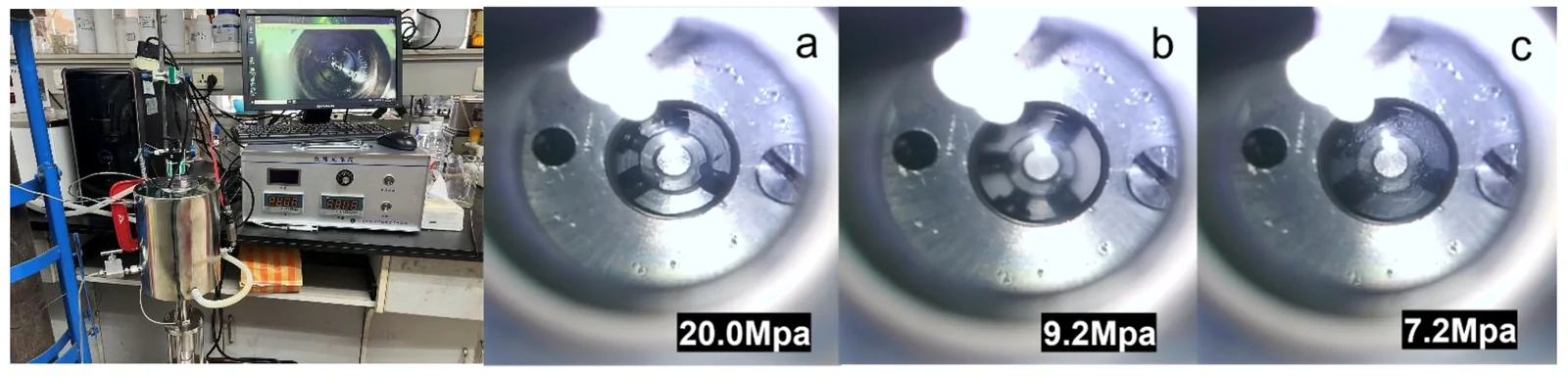
Photographs showing the variable-volume scCO_2_ apparatus and the dissolution states of gas-soluble surfactants at (**a**) 20.0 MPa, (**b**) 9.2 MPa, (**c**) 7.2 MPa.

Figure 1 shows the dissolution state of surfactants in the variable-volume scCO_2_ apparatus. The cloud-point pressure was employed as the key parameter to evaluate the CO_2_-solubility of each surfactant.

As the pressure increased, the CO_2_ in the visual cell gradually transformed from the gaseous state to the supercritical state. When the pressure reached 20 MPa, the gas-soluble surfactant was completely dissolved, and the cell displayed a transparent, uniform single phase (Figure 1a). During depressurization, the miscible state remained unchanged as the pressure was reduced, and the system stayed consistently clear and transparent. When the pressure dropped to the cloud-point pressure, the system in the cell suddenly turned cloudy (Figure 1b), indicating that phase separation occurred in the gas-soluble surfactant/scCO_2_ system and that the surfactant transitioned from a fully dissolved state to a partially dissolved state. As the pressure continued to decrease, the CO_2_ departed from the supercritical state and the surfactant precipitated completely (Figure 1c). These observations directly demonstrate the dissolution and separation processes of the gas-soluble surfactant/scCO_2_ system.

**Figure 2:**
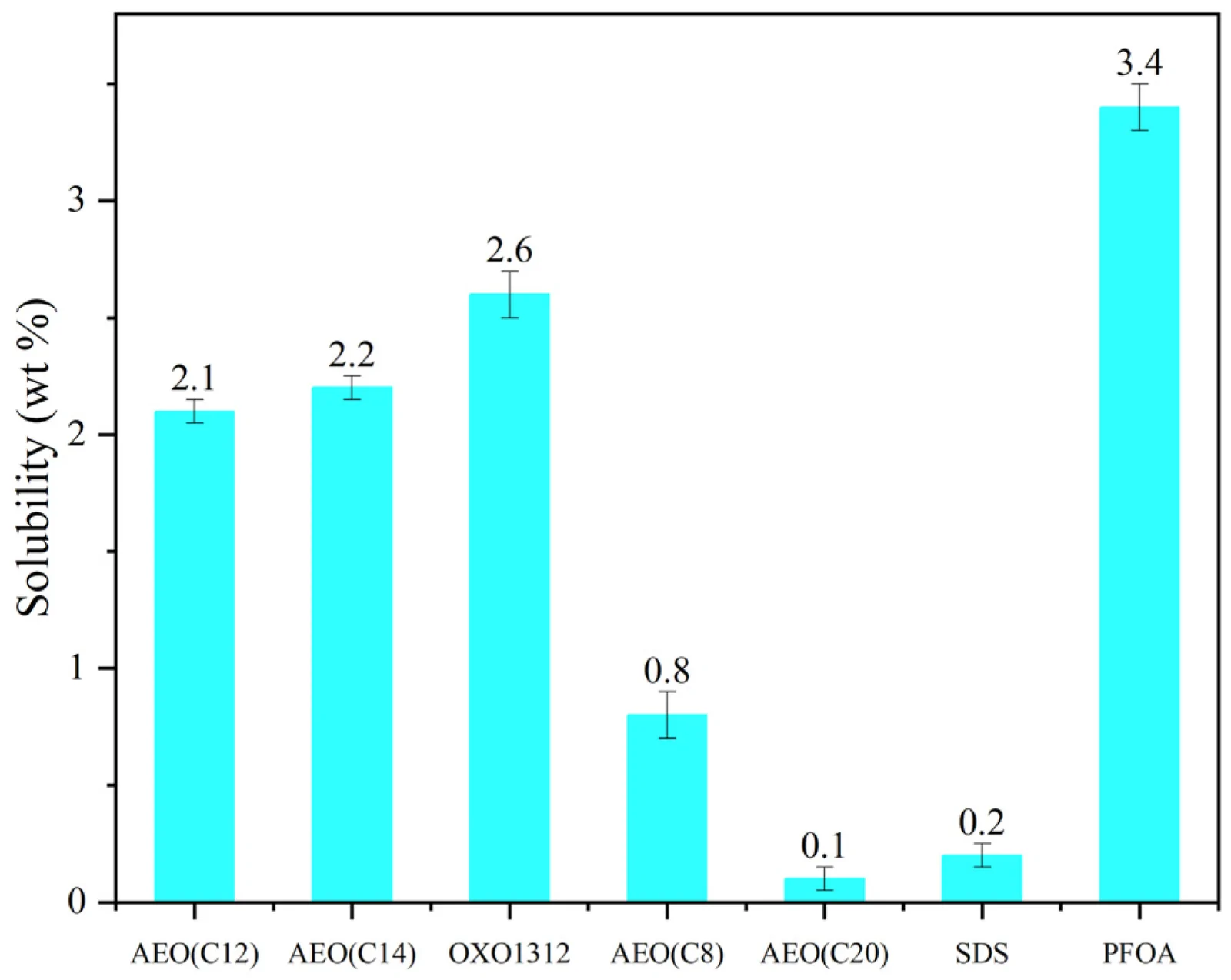
Comparison of scCO_2_ solubilities for different types of surfactants.

Figure 2 compares the scCO_2_ solubilities of different types of surfactants at 60 °C and 20 MPa. The results show that the nonionic surfactants generally exhibit solubilities in CO_2_ higher than 2 wt%, whereas commonly used anionic surfactants such as SDS show solubilities below 1 wt%, indicating that molecules with nonionic ether structures are more favorable for dispersion and dissolution in the scCO_2_ phase. Although the fluorine-containing surfactant PFOA shows the highest solubility (3.4 wt%), nonionic surfactants are more suitable for CO_2_ foam systems when solubility, cost, and potential environmental toxicity are considered together. Meanwhile, surfactants with C8 or C20 chains show sharp decreases in solubility, indicating a clear carbon-chain-length window effect: the C8 chain is too polar, whereas the C20 chain is far more hydrophobic than the other chain lengths, and only an intermediate chain length yields the best CO_2_ compatibility. Consequently, the OXO-series surfactants bearing C13 chains exhibit higher solubilities.

The above solubility differences are closely related to the unique molecular properties of CO_2_. Although CO_2_ possesses a linear symmetric structure with zero net dipole moment, it has a significant quadrupole moment and can interact strongly with certain oxygen-containing functional groups via quadrupole–dipole or quadrupole–hydrogen-bond interactions [11]. Nonionic surfactant molecules contain oxygen-containing groups such as ether (–O–), ester (–COO–), or carbonyl (C=O) groups, whose lone-pair electrons can form specific interactions with the quadrupole moment of CO_2_, thereby markedly enhancing the solubility of the surfactant in the CO_2_ phase. The branched structure further amplifies this solubility advantage: the OXO series (with C13 chains) exhibits considerably higher CO_2_ solubilities than the straight-chain AEO series. This is because the introduction of branched alkyl groups increases the disorder and free volume of the molecular chains, weakening the intermolecular van der Waals aggregation tendency and allowing the surfactant molecules to exist as smaller aggregates (e.g., monomers or small micelles) in the CO_2_ phase. Consequently, the cloud-point pressure is reduced, and the single-phase dissolution region is broadened [12]. Based on the results in Figure 2, the OXO series, which exhibited the highest solubilities, was selected for further investigation of the effects of individual variables on its scCO_2_ dissolution behavior.

### 2.2. Effect of Surfactant Hydrophilicity–Lipophilicity on Solubility

**Figure 3:**
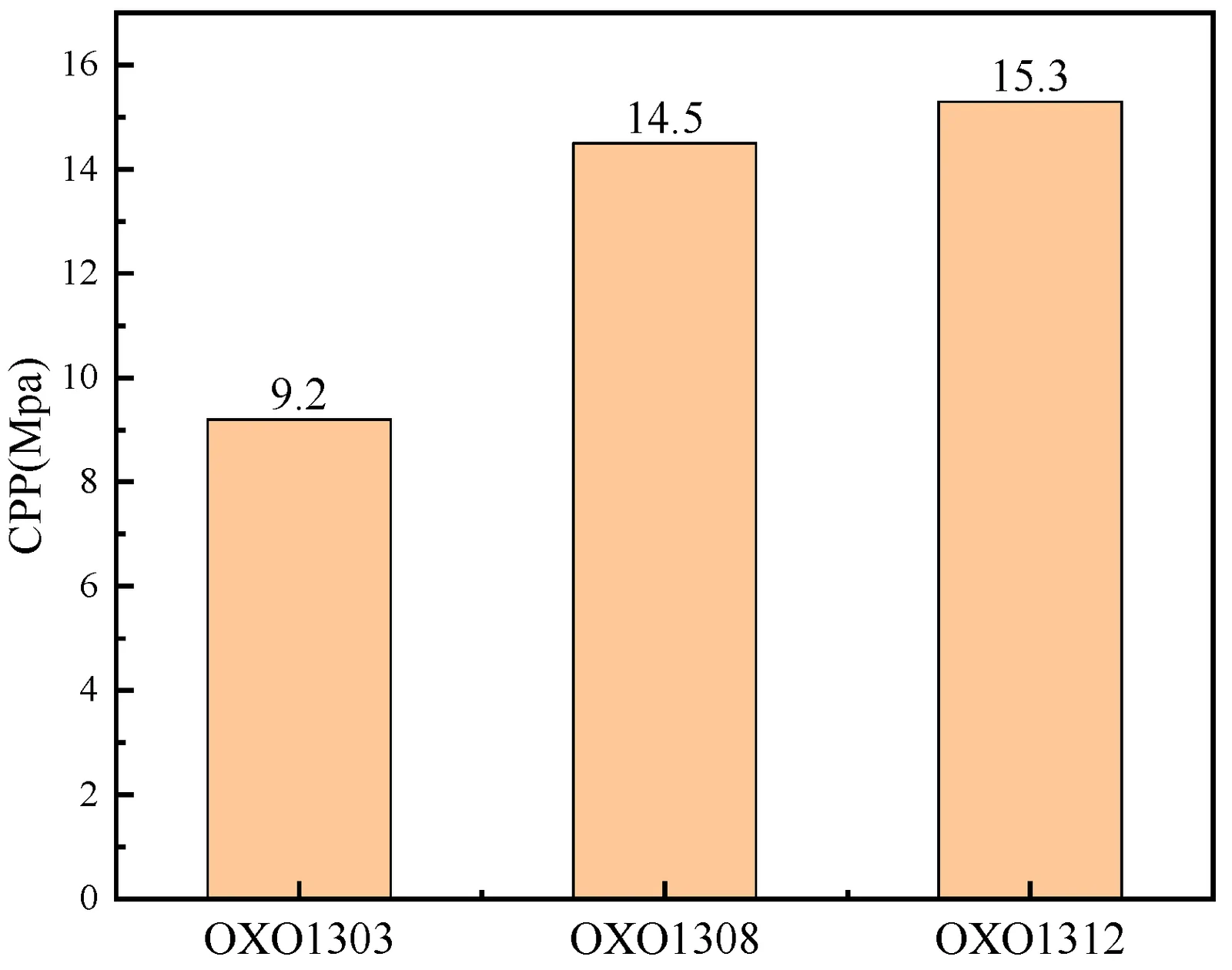
Effect of surfactant hydrophilicity–lipophilicity on solubility in scCO_2_.

The solubilities of the OXO-series surfactants (with different numbers of hydrophilic units) in scCO_2_ were measured at 40 °C (Figure 3). With an increasing number of hydrophilic units, the cloud-point pressure of the OXO surfactants showed an upward trend. When *n* = 3 (OXO1303), the cloud-point pressure was below 10 MPa—the lowest among all tested samples—indicating that OXO1303 has the best CO_2_ solubility. When n was increased to 8 (OXO1308), the cloud-point pressure increased, and upon further increasing n to 12 (OXO1312), the cloud-point pressure reached 15.3 MPa, which is still below the experimental pressure of 20 MPa.

This trend can be explained from the perspectives of molecular configuration and CO_2_ interactions: the ether oxygen atoms (–O–) within the polyoxyethylene chain can interact specifically with CO_2_ molecules, constituting the structural basis of the CO_2_-philicity of nonionic surfactants. However, increasing the length of the hydrophilic chain raises the molecular polarity and reduces the compatibility of the surfactant with the nonpolar CO_2_ phase. At the same time, longer hydrophilic chains are prone to forming intermolecular hydrogen bonds, leading to molecular aggregation, which in turn requires higher pressures to maintain the single-phase dissolution state [17]. Therefore, shorter hydrophilic chains (*n* = 3), with moderate polarity and weak aggregation tendencies, yield the lowest cloud-point pressure and the best CO_2_ solubility.

It is worth noting that even when the hydrophilic chain length is extended to *n* = 12, the cloud-point pressure of OXO1312 (15.3 MPa) remains below 20 MPa, and the larger number of hydrophilic units makes the molecule more readily miscible with water. This result indicates that the OXO-series surfactants possess good CO_2_ solubility over a broad range of hydrophilic chain lengths, demonstrating their great potential as additives for CO_2_ mixed fluids.

### 2.3. Effect of Ambient Temperature on Solubility

**Figure 4:**
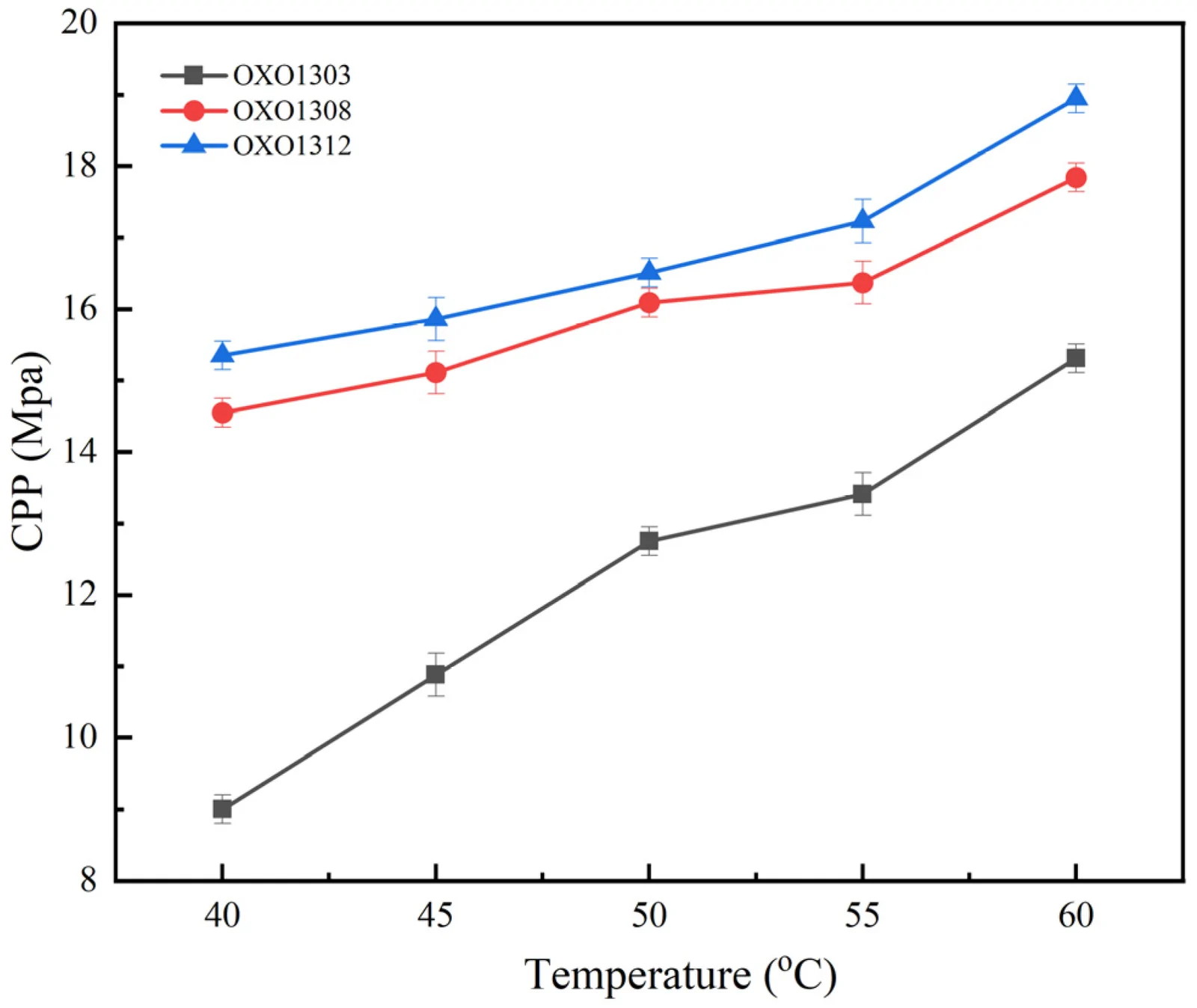
Cloud-point pressures of OXO-series surfactants at different temperatures.

Within the temperature range of 40–60 °C, the temperature exerts a pronounced effect on the cloud-point pressure: the cloud-point pressures of all OXO-series surfactants in CO_2_ increase with rising temperature (Figure 4). This phenomenon is attributed to the decrease in CO_2_ density with increasing temperature, which weakens the intermolecular interactions and reduces the dissolving capacity. In addition, the free volumes of the CO_2_ solvent molecules and the polyether molecules expand to different extents upon heating, leading to a continuously increasing difference in cohesive energy density, which hampers dissolution and raises the cloud-point pressures of the OXO-series surfactants in CO_2_.

Compared with OXO1308 and OXO1312, the temperature exerts a greater influence on OXO1303: over a 20 °C temperature span, the cloud-point pressure of OXO1303 increases by 70.1%, whereas the increments for the other two surfactants do not exceed 30%. The cloud-point pressures of the surfactants with long hydrophilic chains are less affected by temperature, because the intensified molecular thermal motion at elevated temperatures reduces the curling and entanglement of the long-chain structures, thereby increasing the probability of interaction between the long chains and CO_2_ and diminishing the adverse effect of temperature on their cloud-point pressures. It should be emphasized that CO_2_ solubility is not the sole criterion for screening gas-soluble foaming agents. The cloud-point pressures of all three OXO-series surfactants are below 20 MPa, confirming that this series can be dissolved in scCO_2_. However, a higher CO_2_ solubility corresponds to fewer hydrophilic EO units and thus poorer water-phase compatibility, which is unfavorable for interfacial adsorption and foam generation upon contact with formation water. OXO1312, bearing the largest number of EO units while retaining a cloud-point pressure (15.3 MPa) well below the experimental pressure, offers the best balance between CO_2_-phase transportability and water-phase compatibility, and was therefore selected as the foaming agent for the subsequent foam-performance evaluation.

### 2.4. Foaming Performance of the Binary Gas-Soluble Surfactant Systems

#### 2.4.1. Effect of Gas-Soluble Surfactant Type on Drainage Half-Life

**Figure 5:**
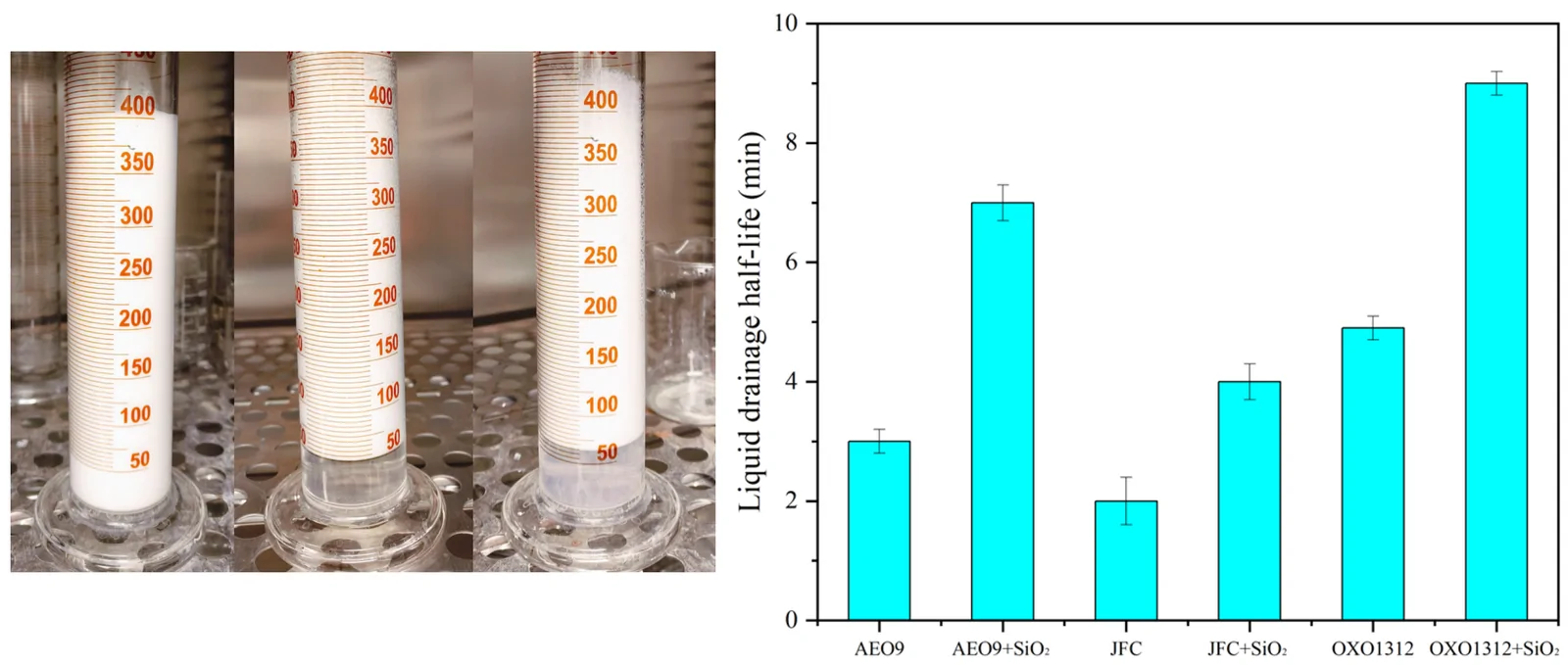
Effects of nonionic surfactant type and nano-SiO_2_ on drainage half-life.

Figure 5 compares the drainage half-lives of three foaming systems (0.8 wt% nonionic surfactant) under ambient temperature and pressure in a CO_2_ atmosphere with deionized water. From the perspective of molecular structure, JFC, with its short hydrophobic carbon chain, forms a thin interfacial adsorption layer with weak intermolecular interactions and low liquid-film mechanical strength, leading to rapid drainage and a short half-life. AEO9, a long-chain fatty alcohol polyoxyethylene ether, has a straight hydrophobic tail that endows the interface with a relatively dense film-packing structure and strong intermolecular van der Waals interactions, thereby forming a relatively compact adsorbed film at the gas–liquid interface. The hydrophobic group of OXO1312 adopts a branched isomeric structure, generating an elastic film with steric hindrance at the gas–liquid interface with CO_2_. Meanwhile, the EO addition number and the hydrophobic chain length of fatty alcohol polyoxyethylene ether nonionic surfactants are the core parameters determining the interfacial film-forming capability [14]. OXO1312 has a higher EO number than the other two surfactants, which enhances its hydrophilicity and improves its interfacial adsorption efficiency. Hence, the drainage half-life of OXO1312 is much longer than those of the other two surfactants.

After 1.5 wt% nano-SiO_2_ was added to each surfactant solution, the half-lives of all three systems were significantly enhanced. This is attributed to the formation of a Pickering-type stabilizing structure at the gas–liquid interface by the nano-SiO_2_ particles, which markedly improves the interfacial viscoelasticity; meanwhile, the bridging and blocking effects of the particles at the Plateau borders suppress rapid liquid drainage [18,19]. Benefiting from its dense basal adsorption film, the AEO9 system provides a favorable substrate for the particles and facilitates the formation of a stable SiO_2_ monolayer at the interface. In addition, the low-pH environment induced by CO_2_ dissolution protonates the silanol groups on the SiO_2_ surface and lowers its surface charge density, which favors the formation of hydrogen bonds between the surface silanol groups and the ether oxygen atoms of the EO chains of the nonionic surfactant [17]; this hydrogen-bonding adsorption anchors the surfactant–particle composite layer at the gas–liquid interface, so that the synergistic enhancement of this system is maintained or even amplified under a CO_2_ atmosphere. In contrast, JFC, with its short hydrophobic chain, forms a thin basal liquid film with insufficient viscoelasticity, and the foam-stabilizing contribution of the particles is limited by the intrinsic interfacial film-forming capacity of the surfactant; moreover, under weakly acidic conditions, the thin liquid film of the JFC system is more susceptible to the pH perturbation caused by CO_2_ dissolution, and consequently the improvement in its drainage half-life is limited.

The OXO1312 + SiO_2_ system exhibits the longest drainage half-life, with an increment of 84%, which originates from the deep synergy between the isomeric branched-chain surfactant and the nanoparticles: the isomeric hydrophobic chain provides an elastic basal liquid film, and the incorporation of SiO_2_ particles further enhances the steric hindrance and mechanical strength on top of the elastic film. Together, they obstruct the liquid-film drainage channels and markedly suppress the gravitational drainage process. Consistent with the drainage half-life results, the foam comprehensive index (FCI) of OXO1312/nano-SiO_2_ (FCI = 2362) was higher than AEO9/nano-SiO_2_ (FCI = 1680) and JFC/nano-SiO_2_ (FCI = 990). In summary, OXO1312 exhibits the best foam stability owing to the high interfacial adsorption efficiency and elastic liquid-film characteristics imparted by its branched hydrophobic chain.

#### 2.4.2. Effect of Gas-Soluble Surfactant Concentration on Drainage Half-Life

**Figure 6:**
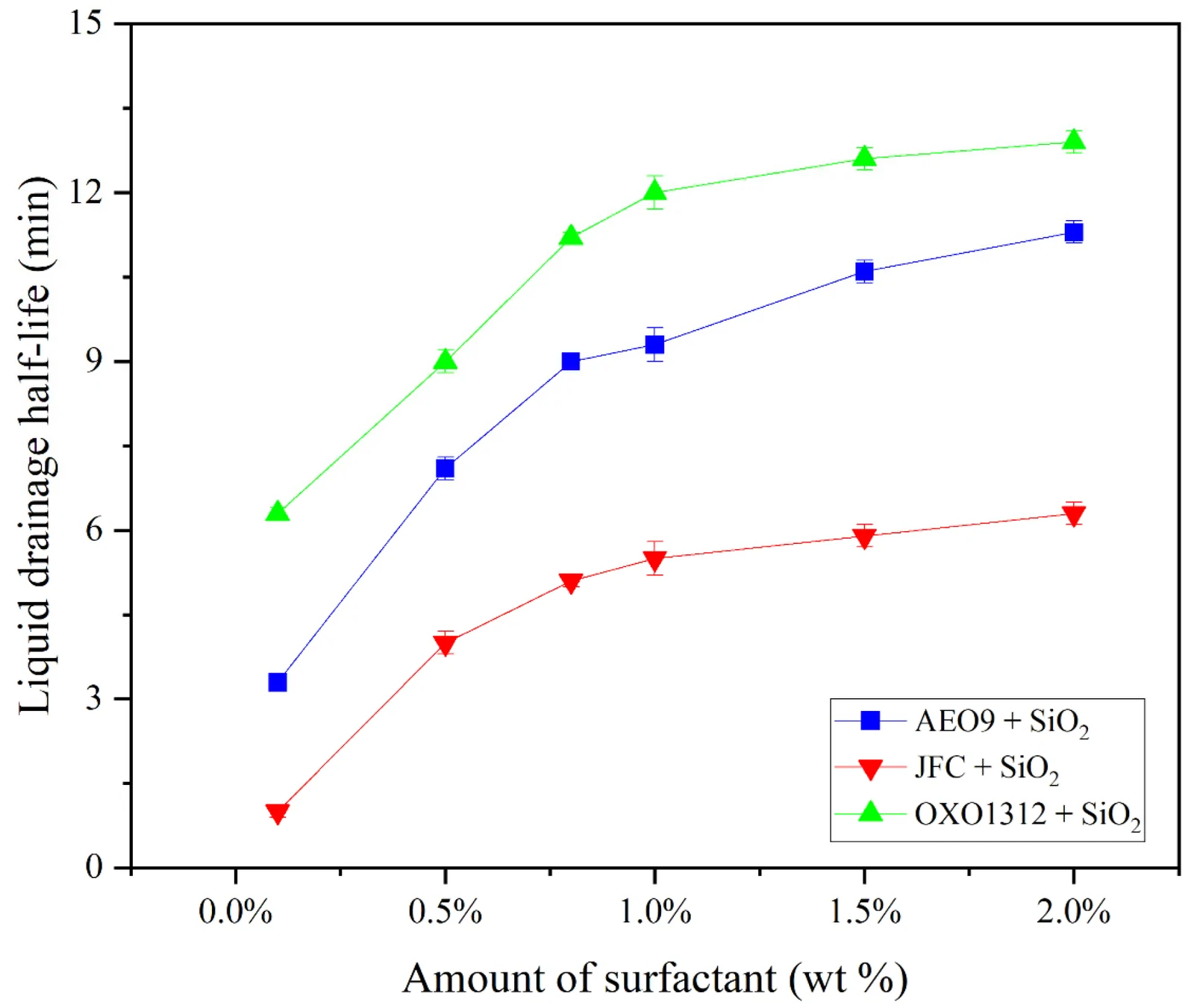
Effect of surfactant concentration on drainage half-life in the binary systems.

Figure 6 shows the influence of the surfactant concentration (0.1–2.0 wt%) on the drainage half-life in three composite systems: AEO9 + SiO_2_, JFC + SiO_2_, and OXO1312 + SiO_2_. With increasing surfactant concentration, all three systems display a trend of a rapid initial rise followed by a plateau in the drainage half-life. In the low-concentration region (0.2–0.8 wt%), the half-life increases significantly with the surfactant concentration, indicating that the adsorption density of surfactant molecules at the gas–liquid interface continuously increases and that the strength and viscoelasticity of the interfacial film are correspondingly enhanced, effectively suppressing liquid drainage at the Plateau borders. Once the concentration exceeds 0.8 wt%, the slopes of the three curves decrease markedly and enter a plateau region, indicating that the interfacial adsorption gradually becomes saturated and that further increases in the surfactant concentration contribute only marginally to extending the foam lifetime. At the same concentration, the OXO1312 + SiO_2_ system exhibits a longer drainage half-life (FCI = 3940) than the other systems (FCI = 2160 for AEO9 and FCI = 1262 for JFC), because the isomeric branched hydrophobic chain of OXO1312 imparts higher interfacial elasticity and steric hindrance, whereas the straight long chain of AEO9 provides a denser film-packing structure and the short chain of JFC results in an insufficient basal liquid-film strength. In addition, the Pickering stabilization of the SiO_2_ nanoparticles shows a pronounced synergy with the surfactant concentration: at low surfactant concentrations, the interfacial adsorption layer is incomplete and the particle-bridging effect is limited; as the concentration increases, the surfactant provides a more stable adsorption substrate for the particles and the particle network becomes denser, so that the synergistic enhancement gradually emerges and reaches a steady level at high concentrations. In summary, under ambient temperature and pressure in a CO_2_ atmosphere, a surfactant concentration of approximately 0.8 wt% is sufficient to construct a relatively stable interfacial film–particle composite network in all three systems; further increases in concentration can only slightly extend the half-life while yielding substantially diminishing economic returns.

#### 2.4.3. Effect of Nano-SiO_2_ Loading in the Binary Gas-Soluble Surfactant System

**Figure 7:**
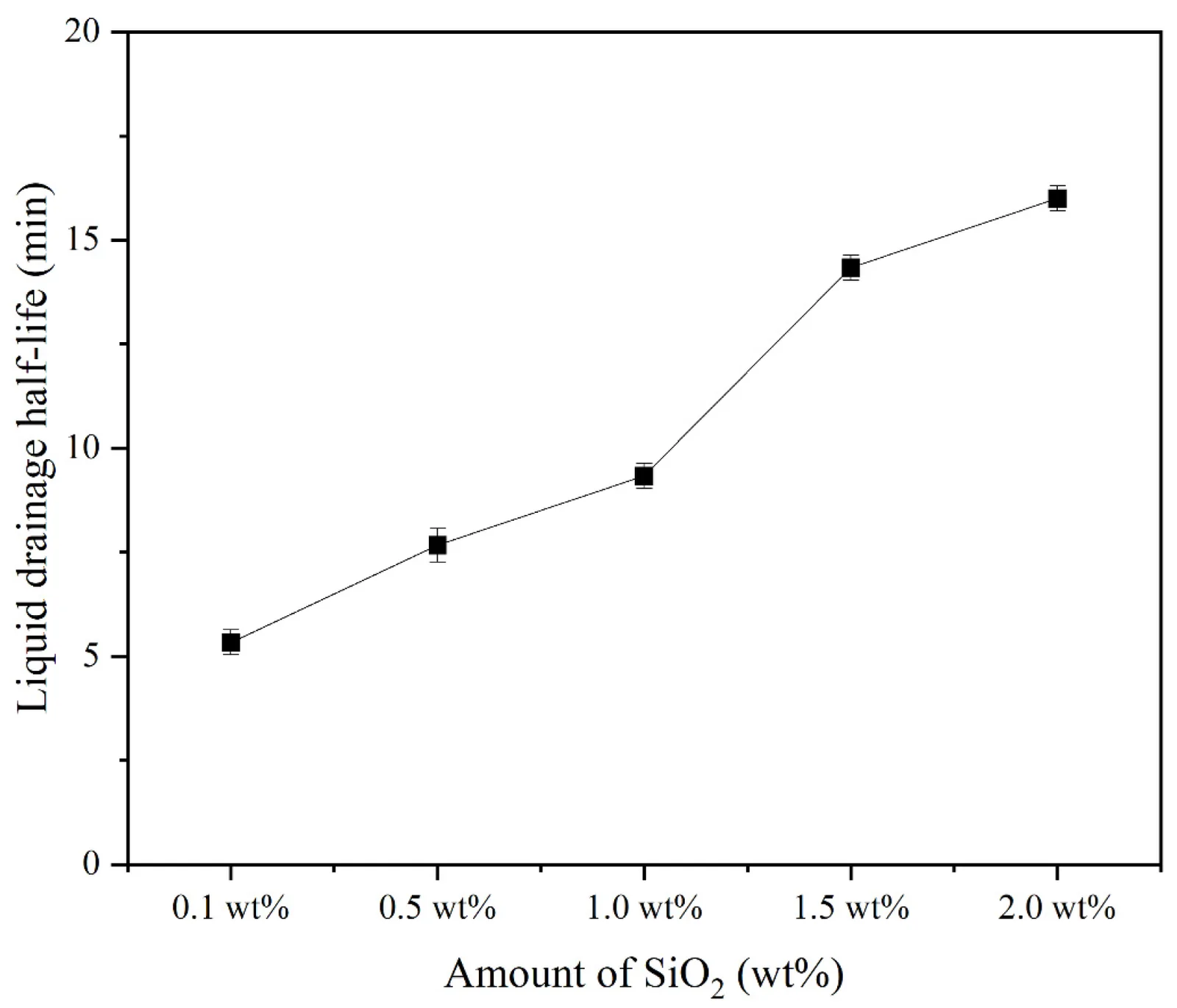
Effect of nano-SiO_2_ mass fraction on drainage half-life in the OXO1312/nano-SiO_2_ binary system.

Under ambient temperature and pressure in a CO_2_ atmosphere with deionized water, 0.1–2.0 wt% nano-SiO_2_ was added to a 0.8 wt% OXO1312 foaming system, and the drainage half-lives of the resulting systems are shown in Figure 7.

Figure 7 shows that as the nano-SiO_2_ content increases from 0.1 to 2.0 wt% in the binary system, the drainage half-life continues to rise significantly but with a clear nonlinear growth pattern. In the 0.1–1.0 wt% range, the drainage half-life increases only slowly, indicating that the nano-SiO_2_ particles gradually accumulate on the composite adsorption layer constructed jointly with OXO1312 and form incomplete Pickering particle clusters, which extend the foam lifetime by enhancing the interfacial viscoelasticity and blocking the liquid-drainage channels at the Plateau borders. When the nano-SiO_2_ content exceeds 1.0 wt%, the drainage half-life jumps markedly, indicating that the average inter-particle distance has shortened sufficiently to form a continuous and dense Pickering network; the interfacial yield stress increases substantially, and the liquid film becomes significantly more resistant to capillary-pressure drainage and CO_2_ trans-membrane diffusion. At 2.0 wt%, the increase in the drainage half-life slows down noticeably, indicating that the interfacial particle packing is approaching saturation. In summary, for the OXO1312/nano-SiO_2_ binary system, 1.5 wt% SiO_2_ can be regarded as the optimal loading that balances foam-stabilizing performance with economic efficiency.

#### 2.4.4. Effects of Other External Conditions on the Drainage Half-Life

**Figure 8:**
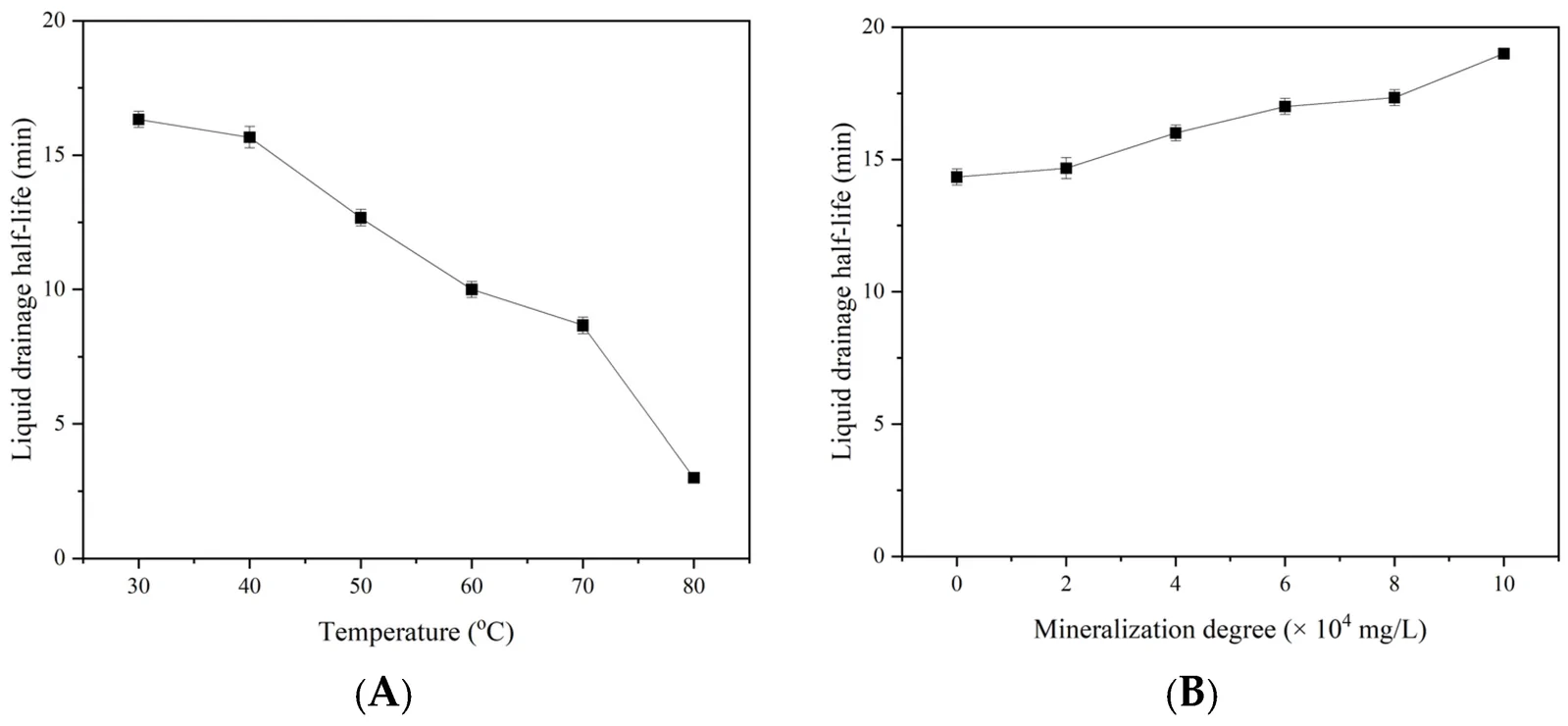
Effects of other external conditions on the drainage half-life of the binary system: (**A**) effect of temperature; (**B**) effect of aqueous salinity.

Under a CO_2_ atmosphere with deionized water, the drainage half-lives of the OXO1312/nano-SiO_2_ (0.8 wt%/1.5 wt%) binary foaming system at different temperatures are shown in Figure 8A.

As shown in Figure 8A, the drainage half-life of the system decreases significantly with increasing temperature; the decrease is slow below 50 °C but becomes sharp above 70 °C. First, the elevated temperature reduces the viscosity of the liquid phase and intensifies molecular thermal motion, directly accelerating gravitational drainage. Second, the temperature rise reduces the adsorption density of the surfactant at the gas–liquid interface and thins the hydration shell of the EO chain segments, leading to a simultaneous decrease in the mechanical strength and viscoelasticity of the liquid film. Finally, the high temperature disrupts the hydrogen-bond interactions between the nano-SiO_2_ particles and the surfactant, causing the Pickering network to loosen or even locally disintegrate. When the temperature exceeds 70 °C, the nonionic surfactant becomes insoluble and precipitates owing to the cloud-point phenomenon, losing its foaming capability.

The drainage half-lives of the OXO1312/nano-SiO_2_ (0.8 wt%/1.5 wt%) binary foaming system in aqueous solutions with different salinities are shown in Figure 8B. The drainage half-life gradually increases with rising salinity. This phenomenon is primarily attributed to the compression of the interfacial electrical double layer by the electrolyte: at higher salinity, the electrostatic repulsion within the system is weakened, and the interfacial adsorption layer becomes more closely packed. At the same time, the electrolyte compresses the electrical double layer of the SiO_2_ particles and reduces their surface charge density, making the particles more hydrophobic and more readily adsorbed at the gas–liquid interface to form a denser Pickering network. In addition, the decrease in water activity and the thinning of the hydration layer at high salinity may also slow liquid-film drainage to some extent.

### 2.5. Microstructure and Pore-Scale Dynamic Evolution of the Binary-System Foam

#### 2.5.1. Foam Microscopic Morphology and Bubble-Size Distribution

**Figure 9:**
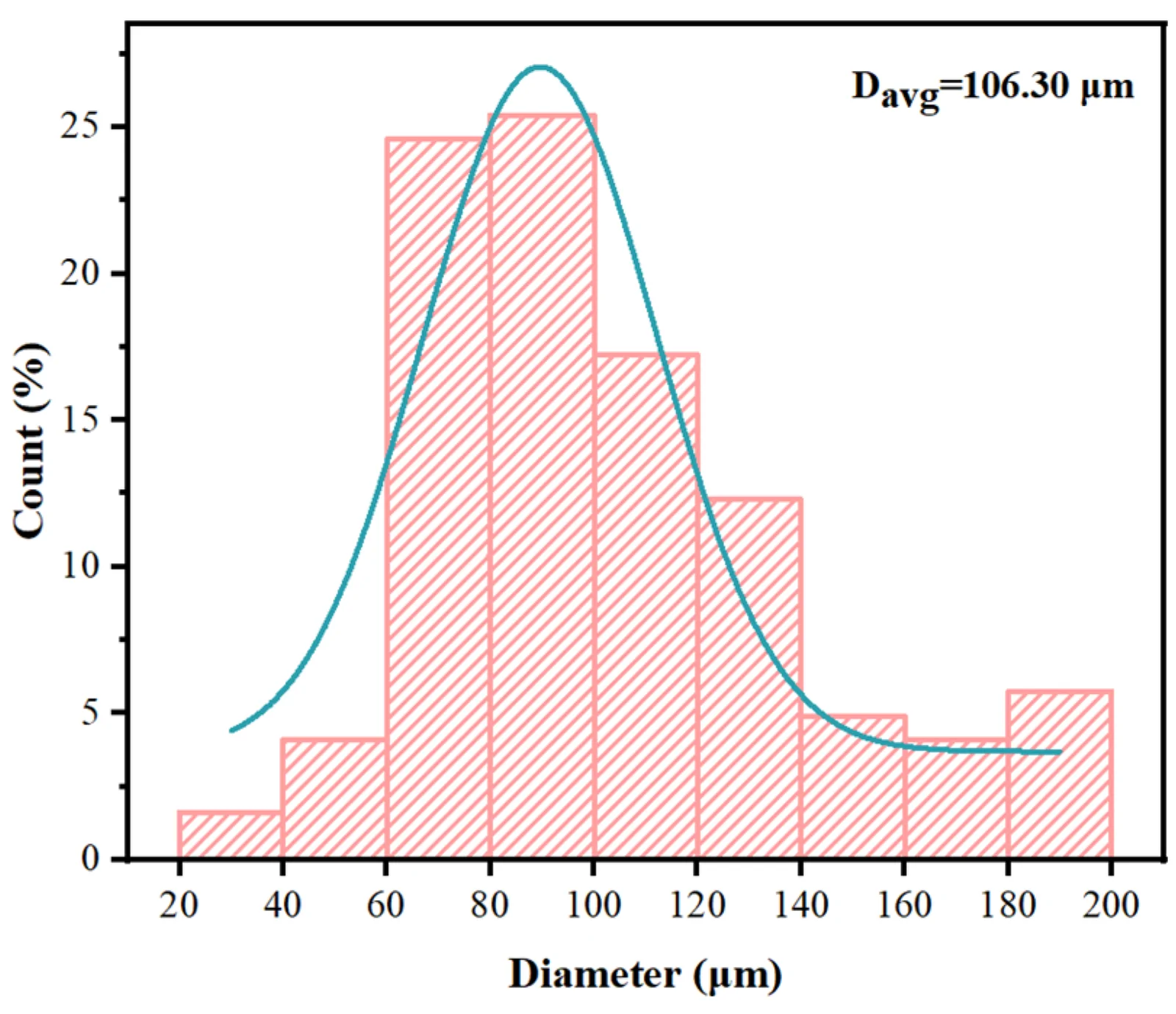
Statistical distribution of bubble diameters in the binary system.

Figure 9 presents the statistical distribution of bubble diameters in the binary system after foaming. The bubble-diameter distribution follows an approximately unimodal and quasi-normal pattern with a pronounced positive skew (right tail): the average diameter D is approximately 106.30 μm, the main peak is located in the 80–100 μm range, and bubbles with diameters greater than 140 μm are noticeably fewer. From the perspective of the foam-stabilization mechanism, this distribution indicates that the binary system can generate fine- to medium-sized bubbles dominated by sizes around 100 μm under a CO_2_ atmosphere. The smaller average bubble size implies a higher specific surface area and a relatively uniform Laplace pressure distribution, which is conducive to reducing the driving force for Ostwald ripening. However, a certain number of large bubbles (>140 μm) appear in the tail, and these large bubbles continuously grow by absorbing CO_2_, thus becoming a potential weak point in the long-term stability of the system. Meanwhile, the Pickering network formed by the nano-SiO_2_ particles within the binary system effectively suppresses bubble coalescence, keeping the bubble-size distribution relatively stable. In summary, the binary system exhibits a relatively fine foam structure, consistent with its longer drainage half-life and stronger foam-stabilizing capability. For the bubble-size statistics, at least five fields of view were randomly selected for each sample, and bubbles in total were counted to ensure the statistical representativeness of the measured average diameter and size distribution.

For comparison, the foam morphology and its temporal evolution of 0.8 wt% OXO1312 (without nano-SiO_2_) were also examined under identical conditions.

**Figure 10:**
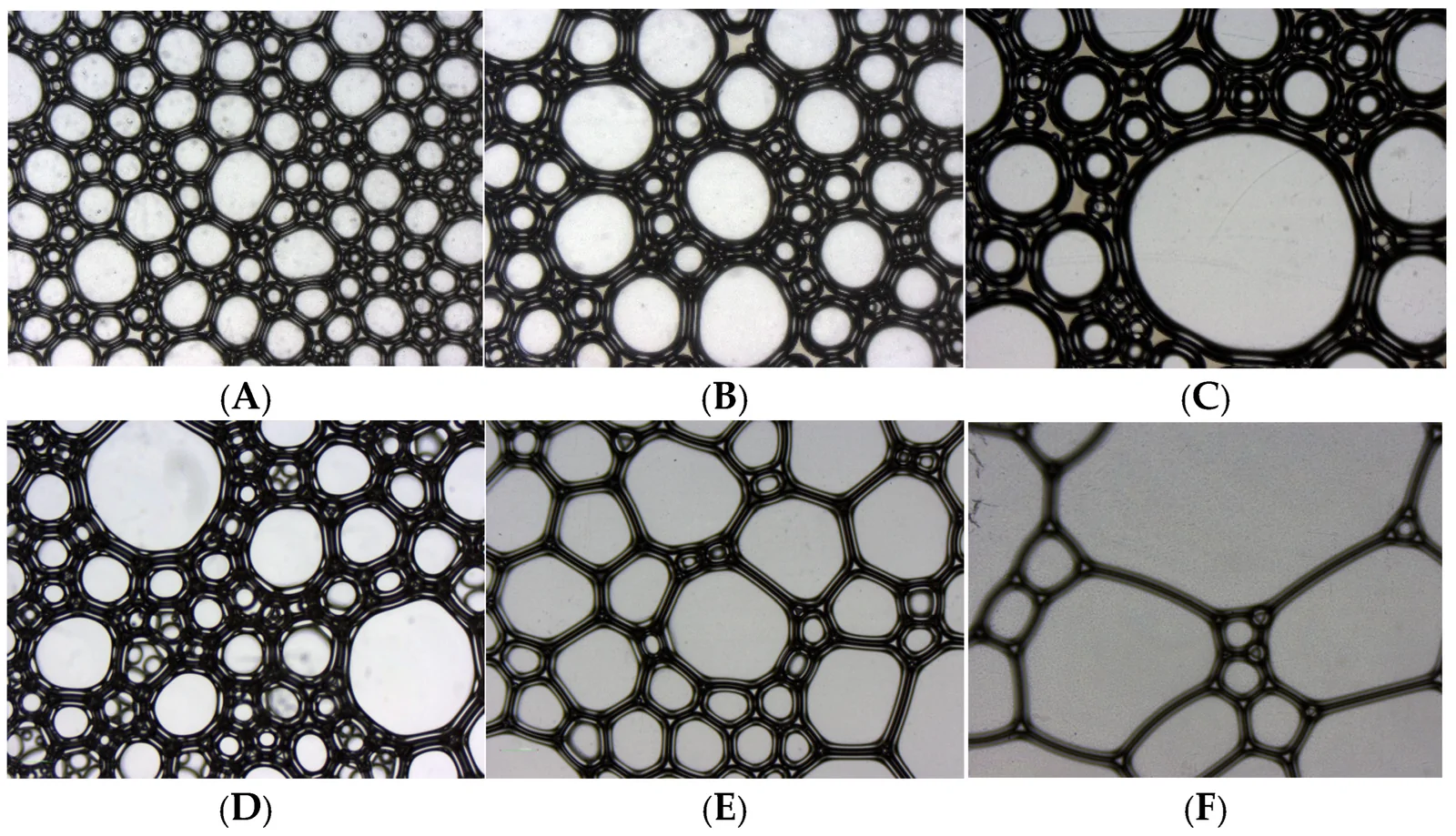
Foam morphology at different times: (**A**) OXO1312/nano-SiO_2_, 5 min; (**B**) OXO1312/nano-SiO_2_, 10 min; (**C**) OXO1312/nano-SiO_2_, 20 min; (**D**) OXO1312, 5 min; (**E**) OXO1312, 10 min; (**F**) OXO1312, 20 min.

Figure 10 shows the microscopic morphologies of the binary-system foam at 5, 10, and 20 min. In the early stage of foaming (5 min, Figure 10A), the foam exhibits a large number of polyhedral structures with relatively uniform sizes; the bubble boundaries are clear and the Plateau borders are thin, with no obvious film rupture or coalescence, indicating that the OXO1312/nano-SiO_2_ composite adsorption-layer network has been initially formed at this stage and effectively inhibits bubble coalescence. As the time extends to 10 min (Figure 10B), a small number of markedly enlarged bubbles begin to appear, accompanied by a decrease in the number of small bubbles and a reduced uniformity of the bubble-size distribution, and some Plateau borders become thicker. These are typical characteristics of Ostwald ripening: driven by the high CO_2_ solubility and the high liquid-film permeability, the CO_2_ within small bubbles diffuses across the liquid film into neighboring large bubbles, causing the large bubbles to grow continuously while the small bubbles shrink or even disappear. At 20 min (Figure 10C), the bubble-size distribution becomes highly non-uniform, with one or several giant bubbles of clearly dominant diameter surrounded by numerous compressed small bubbles, and the Plateau borders become markedly thickened with localized collapse. These observations indicate that, after prolonged evolution, Ostwald ripening dominates; although the Pickering particle network still inhibits bubble coalescence, it cannot fully prevent the coarsening caused by gas trans-membrane diffusion. In summary, the binary system can maintain a relatively uniform fine-bubble morphology on a short time scale, but the bubble coarsening intensifies and the size distribution broadens with prolonged time, which is the main factor limiting its long-term stability.

Figure 10D–F shows the microscopic morphologies of OXO1312 foam at 5, 10, and 20 min. Compared to the binary-system foam, at each observation time the OXO1312 foam displayed larger bubbles and thinner liquid films. Because thinner films drain and rupture more readily and larger bubbles are indicative of more pronounced coarsening and coalescence, this contrast indicates that the binary system yields a foam of higher stability.

#### 2.5.2. Temporal Evolution of Foam Within Micropores

**Figure 11:**
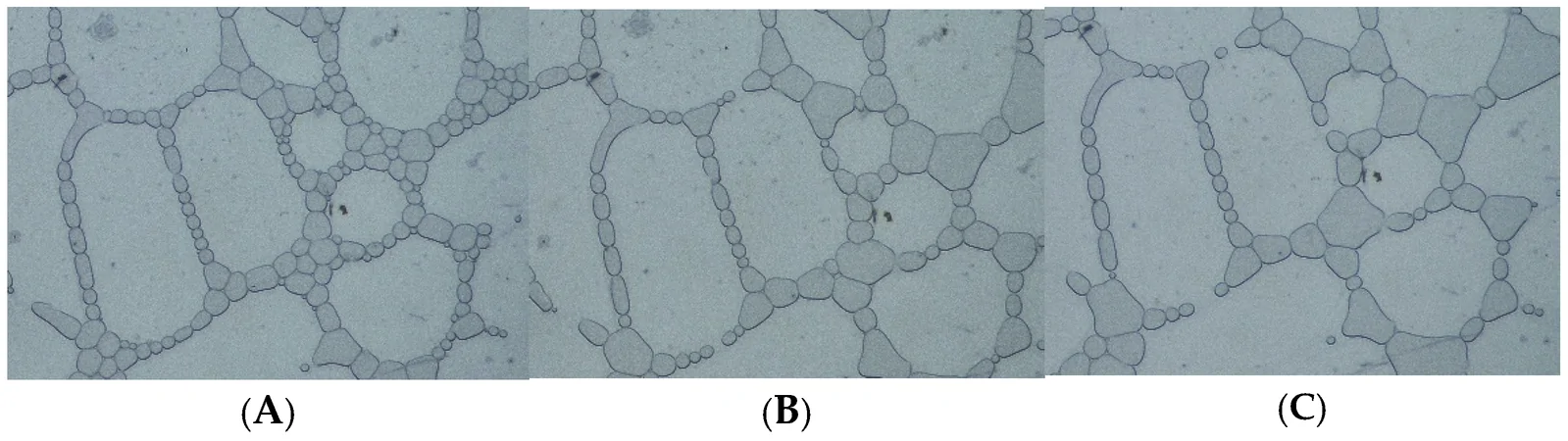
Morphology of foam evolution over time within micropores: (**A**) 5 min; (**B**) 10 min; (**C**) 20 min.

Figure 11 shows the morphological evolution of the binary-system foam within micropores at 5, 10, and 20 min. At 5 min (Figure 11A), the foam in the pores is dominated by small bubbles of relatively uniform size that tightly fill the pores and throats; the liquid films are thin and continuous, with no obvious rupture or connected gas channels, indicating that the binary system can still form a relatively stable foam-blocking structure in confined spaces at this stage, with the OXO1312/nano-SiO_2_ composite system providing effective stabilization for the pore walls and liquid films. As the time extends to 10 min (Figure 11B), the bubble size within the pores increases markedly, the number of small bubbles decreases, and irregular bubble shapes and locally thinned liquid films appear in some regions. This indicates that, driven by the high CO_2_ solubility and the capillary-pressure gradients, the CO_2_ within small bubbles diffuses across the liquid film into large bubbles, causing local bubble coarsening; meanwhile, the shearing and confinement exerted by the pore walls on the bubbles complicate the liquid-film drainage pathways, and some thin liquid films rupture locally under capillary pressure, leading to a small amount of bubble coalescence. At 20 min (Figure 11C), the coarsening of the foam within the pores is further aggravated, and clearly large-bubble-dominant regions appear; the bubble-size distribution becomes highly non-uniform, and in some throats the liquid films rupture to form continuous gas-phase channels, with the foam structure transitioning from a dense plug-flow pattern to a sparse foam. These phenomena indicate that, although the binary system exhibits a relatively long drainage half-life and good Pickering stabilization capability in the bulk phase, the accelerated CO_2_ trans-membrane diffusion, enhanced capillary-pressure effects, and intensified wall-induced liquid-film shear drainage in the confined micropore environment collectively accelerate Ostwald ripening and bubble coalescence. Although SiO_2_ can delay coarsening to a certain extent, it cannot fully inhibit gas channeling during long-term evolution [20].

## 3. Materials and Methods

### 3.1. Materials

Fatty alcohol polyoxyethylene ether surfactants (AEO, JFC, etc., 98%) and isomeric tridecyl alcohol polyoxyethylene ether surfactants (OXO1303, OXO1308, and OXO1312, 98%) were purchased from Youso Chemical Technology Co., Ltd. (Linyi, China). NaCl and organic reagents were purchased from Macklin Biochemical Technology Co., Ltd. (Shanghai, China). Hydrophilic nano-SiO_2_ (particle size of approximately 12 nm, specific surface area of 175–225 m^2^/g was purchased from Wacker Chemicals Co., Ltd. (Shanghai, China). CO_2_ gas (99.9% purity) was purchased from Huaqiang Chemical (Jingzhou, China).

### 3.2. Solubility Test of Gas-Soluble Surfactants in scCO_2_

**Figure 12:**
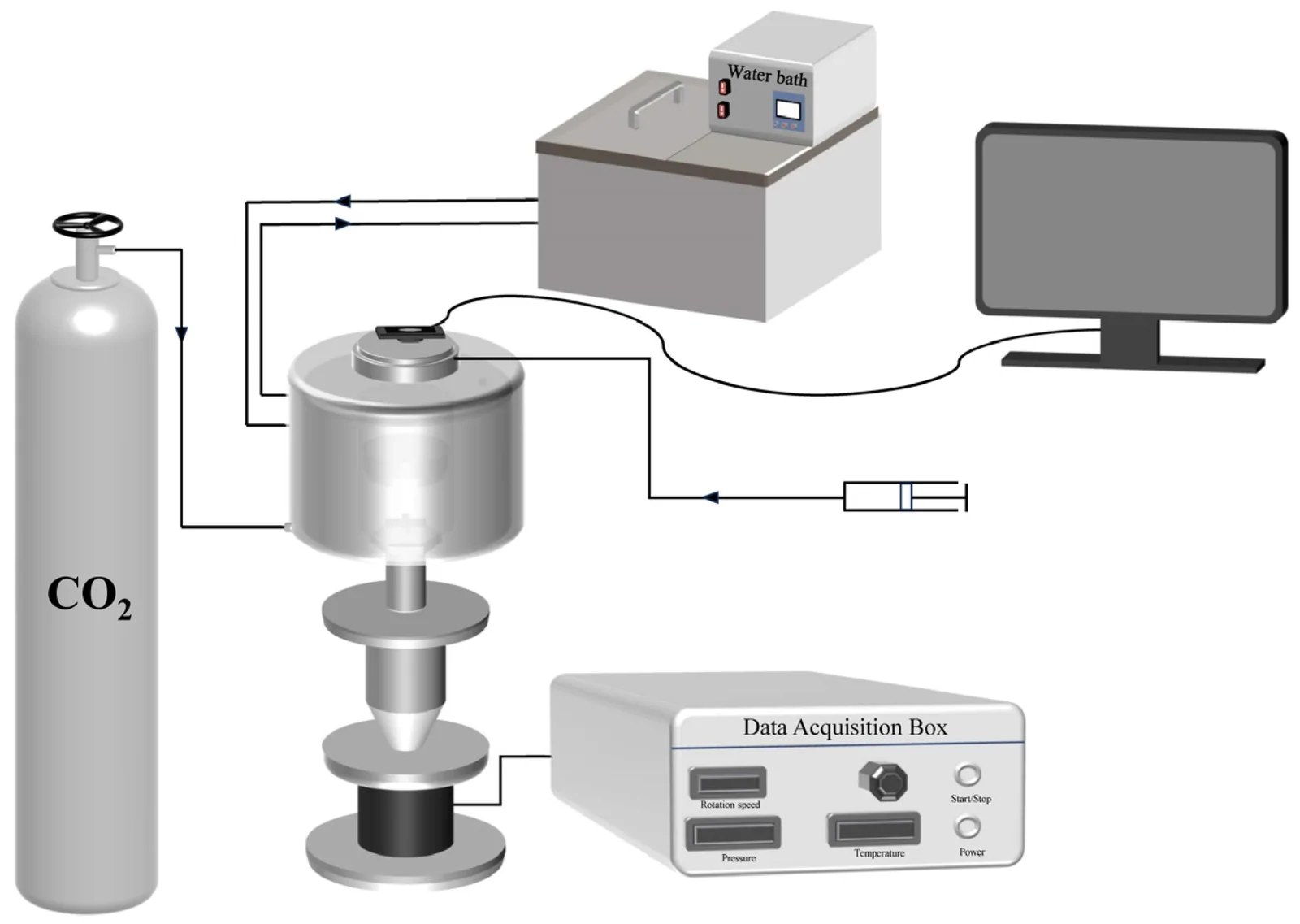
Schematic diagram of the variable-volume supercritical CO_2_ apparatus.

The solubility of the surfactants in CO_2_ was measured using a variable-volume scCO_2_ apparatus (Figure 12). The apparatus consists of a sample cell equipped with a sapphire observation window (maximum volume of 100 cm^3^), a heating system, a magnetic stirrer, and a pressure pump. The temperature of the sample cell was controlled by a temperature controller with an accuracy of ±0.1 °C, and the pressure was measured with a pressure gauge with an accuracy of ±0.001 MPa. A stirring paddle with a rotational speed of 0–1200 rpm was installed at the bottom of the sample cell.

The sample cell was cleaned with acetone and thoroughly dried, after which CO_2_ gas was continuously flushed through the cell for 3 min to expel all air. The surfactant was then added to the sample cell, which was subsequently sealed. Once the cell temperature stabilized at 25 °C, high-pressure CO_2_ gas was introduced. After the pressure stabilized, the temperature was raised to 60 °C, and the cell was pressurized to 20 MPa using the pressure pump. Upon pressure stabilization, the system was stirred at 500 rpm until a transparent homogeneous phase was achieved. The cell pressure was then gradually reduced while the state of the mixed system was monitored through the viewing window. When the system changed from a transparent homogeneous state to a cloudy one, the corresponding pressure was recorded as the cloud-point pressure. Specifically, the cloud-point pressure was objectively identified by monitoring the optical power transmitted through the transparent sample cell during depressurization: a sudden decrease in the transmitted optical power indicates the onset of turbidity, at which the surfactant begins to separate from the CO_2_ medium [21]. Each measurement was repeated at least three times, and the average value was reported. The density of CO_2_ was obtained from the NIST (National Institute of Standards and Technology) fluid properties database. The solubility was calculated using the following equation:

*x* = [*m*/(*ρV* + *m*)] × 100% (1)

where *ρ* (g/cm^3^) is the density of carbon dioxide, *m* (g) is the mass of surfactant added, and *V* (cm^3^) is the volume of the sample cell.

### 3.3. Foaming Performance Evaluation

The CO_2_ foam performance was evaluated using the atmospheric-pressure Waring Blender method. First, 100 mL of a surfactant solution at a given mass fraction was prepared and poured into the stirring vessel. The solution was stirred at 8000 r/min for 3 min under a CO_2_ atmosphere, and the foam volume was immediately measured using a graduated cylinder after the stirring was stopped. The drainage half-life was defined as the time required for 50 mL of liquid, i.e., half of the initial solution volume, to drain from the foam, and the measurement was terminated once the drained liquid reached 50 mL. Each sample was measured at least three times, the average value was reported as the final result, and the error bars in the corresponding figures represent the standard deviations of the replicate measurements. In addition to the drainage half-life, the initial foam volume (V_0_) and the foam comprehensive index (FCI) were used to comprehensively evaluate the foam performance, with FCI = 0.75 × V_0_ × t_1/2_, where t_1/2_ denotes the drainage half-life.

It should be clarified that the atmospheric-pressure Waring Blender tests evaluate the intrinsic foaming ability and foam stability of pre-prepared surfactant solutions in a CO_2_ atmosphere, serving as a convenient formulation-screening approach for mechanism research. The mechanistic understanding obtained from the Waring Blender tests provides a theoretical foundation for subsequent in situ foam generation experiments.

### 3.4. Microscopic Observation of Foam Morphology

The microscopic morphology of the foam and its temporal evolution within micropores were observed using a micro-etched porous-media model replicating the natural pore structure of cores from the Daqing oilfield [22]. The pore network was etched onto a glass plate by photolithography, and the etched plate was bonded to a smooth glass plate to create the microscopic model with a water-wet inner surface. The pore network was etched onto a glass plate by photolithography, and the etched plate was bonded to a smooth glass plate to create the microscopic model. The fabricated micromodel had a porosity of 25%, an average etched depth of approximately 40 μm, and an average pore-body width of approximately 100 μm.

The freshly prepared foam was rapidly injected into the model by a syringe through the inlet port until the pore network was visually filled, and the evolution of the foam morphology was recorded under an optical microscope at ambient temperature and pressure under static conditions. After each experiment, the micromodel was cleaned by successively injecting petroleum ether and ethanol to remove residual contaminants and ensure that all pores and throats were free of contaminants, then dried in a constant-temperature oven at 45 °C for subsequent use.

## 4. Conclusions

In this study, gas-soluble nonionic surfactants (AEO, JFC, and the OXO series) were employed as the research subject, and their dissolution behavior in scCO_2_ was evaluated using a variable-volume scCO_2_ apparatus. Combined with the Pickering stabilization mechanism, the foam-stabilizing performance of the CO_2_ foam flooding system synergistically constructed with nano-SiO_2_ was systematically investigated. The main conclusions are as follows:

(1) The solubilities of the nonionic surfactants in scCO_2_ are significantly higher than those of the anionic surfactants, with the C12–C14 chain-length range exhibiting the best CO_2_ compatibility. OXO1312, falling within the optimal carbon-chain-length range and containing an appropriate number of hydrophilic groups, was selected as the preferred gas-soluble foaming agent.

(2) Under ambient temperature and pressure in a CO_2_ atmosphere, the drainage half-life of the OXO1312/nano-SiO_2_ (0.8 wt%/1.5 wt%) binary system is enhanced by 84% compared with the single OXO1312 system—an increase significantly greater than those of the AEO9/SiO_2_ and JFC/SiO_2_ systems—confirming a deep Pickering synergy between the isomeric branched-chain surfactant and the nanoparticles.

(3) The binary system exhibits favorable stabilizing performance at temperatures below 50 °C and under moderate salinity. The increase in salinity compresses the interfacial electrical double layer, leading to a denser Pickering network structure, which is beneficial to foam stabilization.

(4) It should be noted that the foam performance in this study was evaluated by bulk tests under atmospheric pressure, which reflects the intrinsic foaming ability and stability of the screened formulations but does not directly reproduce the in situ foaming process in porous media; validation of the binary system under porous-medium flow and displacement conditions is therefore required and will be addressed in our future work. Nevertheless, the above results provide theoretical guidance for the formulation design of CO_2_ foam flooding systems for CCUS-EOR processes.

## Data Availability

Data are contained within the article.
